# Nobody Wants to Be Narcan’d: A Pilot Qualitative Analysis of Drug Users’ Perspectives on Naloxone

**DOI:** 10.5811/westjem.2020.10.48768

**Published:** 2021-02-08

**Authors:** Jeffrey T. Lai, Charlotte E. Goldfine, Brittany P. Chapman, Melissa M. Taylor, Rochelle K. Rosen, Stephanie P. Carreiro, Kavita M. Babu

**Affiliations:** * University of Massachusetts Medical School, Department of Emergency Medicine, Worcester, Massachusetts; † Brigham and Women’s Hospital, Department of Emergency Medicine, Boston, Massachusetts; ‡ Brown University School of Public Health, Department of Behavioral and Social Sciences, Providence, Rhode Island

## Abstract

**Introduction:**

Bystander naloxone distribution is an important component of public health initiatives to decrease opioid-related deaths. While there is evidence supporting naloxone distribution programs, the effects of increasing naloxone availability on the behavior of people who use drugs have not been adequately delineated. In this study we sought to 1) evaluate whether individuals’ drug use patterns have changed due to naloxone availability; and 2) explore individuals’ knowledge of, access to, experiences with, and perceptions of naloxone.

**Methods:**

We conducted a pilot study of adults presenting to the emergency department whose medical history included non-medical opioid use. Semi-structured interviews were conducted with participants and thematic analysis was used to code and analyze interview transcripts.

**Results:**

Ten participants completed the study. All were aware of naloxone by brand name (Narcan) and had been trained in its use, and all but one had either currently or previously possessed a kit. Barriers to naloxone administration included fear of legal repercussions, not having it available, and a desire to avoid interrupting another user’s “high.” Of the eight participants who reported being revived with naloxone at least once during their lifetime, all described experiencing a noxious physical response and expressed a desire to avoid receiving it again. Furthermore, participants did not report increasing their use of opioids when naloxone was available.

**Conclusions:**

Participants were accepting of and knowledgeable about naloxone, and were willing to administer naloxone to save a life. Participants tended to use opioids more cautiously when naloxone was present due to fears of experiencing precipitated withdrawal. This study provides preliminary evidence countering the unsubstantiated narrative that increased naloxone availability begets more high-risk opioid use and further supports increasing naloxone access.

## INTRODUCTION

### Background

Bystander naloxone distribution is an evidence-based public health intervention.^1^,^2^ The Surgeon General of the United States has emphasized the importance of the opioid overdose reversal agent, stating succinctly and unambiguously, “knowing how to use naloxone and keeping it within reach can save a life.”^3^ However, efforts to enhance naloxone availability have been hampered by stigma surrounding opioid use disorder (OUD), cost and availability issues, and the unproven assertion that naloxone increases high-risk drug use.^4^–^7^ To evaluate perceptions of naloxone uptake and use in our population, we piloted a semi-structured interview developed in conjunction with the National Drug Early Warning System (NDEWS) workgroup.^8^

### Importance

Although studies have demonstrated that increased availability of naloxone has reduced the rate of opioid overdose fatalities in some communities, there is a paucity of data on whether it has also impacted drug use behaviors. Data regarding knowledge of and attitudes toward naloxone among people who use drugs (PWUD), and the impact of naloxone availability on drug-use behaviors, are urgently needed. In this pilot study, we explored the knowledge and perceptions of naloxone among PWUD in order to obtain more nuanced data to guide public health interventions aimed at decreasing opioid overdose deaths.

### Goals of This Investigation

This study sought to 1) explore individuals’ knowledge of, access to, experiences with, and perceptions of naloxone; and 2) characterize reported changes in individuals’ drug use patterns and attitudes as a result of naloxone accessibility.

## METHODS

### Study Design and Setting

This pilot study was part of a larger multisite effort by the NDEWS workgroup to validate a qualitative interview agenda regarding knowledge and perceptions of bystander naloxone among PWUD. During the trial period (March–April 2019), we enrolled a convenience sample of 10 adult patients who presented to the University of Massachusetts Memorial Medical Center emergency department (ED) with an opioid-related chief complaint (eg, drug overdose, cutaneous abscess, etc) and history of non-medical opioid use. Among the three sites in the study, ours was distinct in that we focused on individuals who presented for evaluation in the emergency care setting rather than in an outpatient clinic. This protocol was approved by the University of Massachusetts Medical School Institutional Review Board. A Certificate of Confidentiality was obtained to provide an additional layer of participant protection.

### Selection of Participants

Study investigators screened the electronic health record ED tracking board for individuals meeting inclusion criteria and approached them once they were deemed medically stable by the provider overseeing their clinical care. Eligible participants were 18–65 years of age, had presented to the ED with an opioid-related chief complaint, had a history of non-medical opioid use, were English-speaking, and were able to provide informed consent. Individuals were excluded if they had previously participated in this study or were in police custody. A study investigator obtained verbal informed consent from participants, who were brought to a private room in the ED for the duration of the interview. Participants were compensated for their time with a $10 gift card to a local retail store.

**Population Health Research Capsule**

What do we already know about this issue?

Bystander naloxone is an evidence-based public health intervention. Increasing naloxone availability is a cornerstone of efforts to combat opioid overdose deaths.

What was the research question?

How is bystander naloxone perceived by opioid users? Has naloxone availability affected opioid use behaviors?

What was the major finding of the study?

Participants were familiar with naloxone and did not report increased opioid use when naloxone was available.

How does this improve population health?

This study affirms that bystander naloxone is acceptable to its intended audience and suggests that naloxone availability does not increase high-risk opioid use.

### Interventions

Two investigators were present during study interviews, with one taking the lead role as facilitator and the other functioning as a notetaker. Investigators administered a brief questionnaire regarding demographic characteristics, as well as a semi-structured interview developed by the NDEWS workgroup, which contained open-ended questions regarding naloxone. A written agenda was used to guide each interview, ensuring that the same key questions were asked of all participants. This allowed each individual to answer in his/her own words, and to describe relevant experiences. The agenda included questions about access to, knowledge of, attitudes about, and experiences with naloxone, as well as each participant’s prior history of drug overdose. Participants were asked to respond based on their own thoughts and experiences, as well as provide insight on their perceptions of what other people who use opioids think about naloxone, and whether the availability of naloxone has changed how other users conceptualize drug use.

### Analysis

We tabulated and entered demographic data into Research Electronic Data Capture (REDCap), a secure web-based application for building and managing online surveys and databases.^9^,^10^ Semi-structured interviews were audio-recorded on a digital voice recorder and transcribed by trained study staff or by a transcription service compliant with the Health Insurance Portability and Accountability Act. Study staff reviewed each transcription to ensure accuracy and to deidentify qualitative data.

Two researchers (BC and MT) independently coded the first two transcripts, creating deductive codes based on questions in the semi-structured qualitative agenda and inductive codes for emergent topics raised by participants. The initial codes were reviewed by the research team, resulting in a preliminary thematic coding scheme. This framework was applied to all transcripts, which were independently coded by both researchers (BC and MT). New codes were created as needed when adjustments were made to accommodate topics in subsequently coded transcripts, which were then retroactively applied to initially coded transcripts as well. Upon completion of independent coding, both researchers met to review differences in coding, which were discussed and refined until agreement between the researchers was reached. After five interviews no further changes were made to the codes. We entered the agreed-upon codes into NVivo 12 Plus (QSR International, Burlington, MA) to complete the thematic analysis, and then reviewed them in aggregate to create summaries of key topic areas.

## RESULTS

A total of 28 individuals were screened for recruitment during the study period. Of those, 12 were unable to be enrolled as they either eloped (n = 4), were unable to provide consent (n = 1), had no non-medical opioid use in the prior six months (n = 6), or reason was not documented (n = 1). Of the 16 potential participants who were approached, six declined to participate in the study: three identified as female and three as male, and ages ranged from 28–35 years with a median age of 32 years. Ten participants were enrolled in this study; the demographics of the study participants are detailed in Table 1. The sample was predominantly young, White males who had been in treatment for OUD on at least one occasion. The majority had previously received naloxone. The sample varied on education, employment, and housing status.

Analysis of semi-structured interviews revealed several themes, which are described in detail below. Additional illustrative quotations are included for each theme (Table 2).

### Familiarity with Narcan (mechanism and use)

All participants were familiar with the brand name “Narcan,” but some were not familiar with the generic term “naloxone.” One individual mistook naloxone for naltrexone. A single participant had never heard the term “naloxone” before. All participants reported having formal naloxone training from sites including local treatment facilities and harm-reduction organizations. Most reported first hearing about naloxone through treatment programs (eg, detox, Alcoholics Anonymous meetings) or correctional facilities, from other people who use opioids for non-medical purposes, and from occasions where they had received it for overdose reversal. Two participants informed study staff that they could not recall how they first learned about naloxone because they had “known about it for so long.”

Most participants understood the general purpose of naloxone to be reversing an opioid overdose, yet there were varying degrees of knowledge about the exact underlying mechanisms. The majority of participants used specific terminology implying blockade or antagonism when describing how naloxone works (eg, “receptor,” “blocker,” and “reversant”). Most participants identified naloxone’s specificity for opioids, but there were two participants who also questioned its utility for other substances, such as alcohol.

All but one participant reported that they currently or previously possessed a naloxone kit. Of those nine, three participants reported that their reasoning for carrying a kit was to save the lives of others. One participant stated, “If someone needed it, I would rather have it than be powerless.” The majority of participants reported obtaining naloxone kits that contained the newer, “easy” plunger-style nasal spray. Three participants mentioned that they had previously obtained the more “difficult to use” older version that required assembly.

### Naloxone Is Available and Easy to Obtain

Participants universally agreed that naloxone kits were available and easy to obtain from a variety of organizations (eg, pharmacies, treatment facilities). All participants knew the process for obtaining a naloxone kit, and several reported obtaining it from a harm-reduction agency (eg, needle exchange) that distributed it for free and provided training. When asked how programs that distribute naloxone could improve their services, some participants suggested increasing access by providing naloxone kits by default whenever someone visits a needle exchange or leaves a treatment program, and by implementing mobile programs of outreach workers to distribute it within the community.

### Naloxone Availability Is Viewed Positively

Participants perceived naloxone as a life-saving drug and were thankful for its presence in the community. One participant stated, “[I] think it’s an amazing drug. I’ve seen it save people’s lives.” Some participants reported feeling empowered by carrying naloxone and said they would use it to revive someone. When asked how individuals who had been revived by naloxone were perceived by other people who use opioids, many participants responded by saying they were “lucky.” Some participants stated that they themselves felt lucky after being revived with naloxone.

### Naloxone Produces Aversive Symptoms During Reversal

All participants who had previously been revived with naloxone reported experiencing extraordinarily unpleasant physical responses consistent with severe opioid withdrawal (eg, nausea, vomiting, diffuse body pain). One participant described it as the worst pain he had ever experienced. When these participants were asked about their emotional response, several disclosed that they felt embarrassed or experienced feelings of depression and anxiety regarding their return to opioid use. Participants acknowledged that receiving naloxone was an experience that they would go to great lengths to avoid. However, in the event that they were to overdose and require naloxone to save their life, they hoped someone would administer it.

### Availability of Naloxone Does Not Increase Risky Drug-use Behavior

Participants were unanimous that their decision to use opioids did not depend on naloxone availability. While participants speculated vaguely that a hypothetical “other” group of people who use opioids might adopt riskier drug use behavior due to the availability of naloxone (such as taking bigger doses or using more often), all participants explicitly denied that they themselves engaged in riskier behavior and/or increased their opioid use in any way due to the availability of naloxone. Several participants reported that they had recently experienced a return-to-use event, but none identified naloxone availability as playing any role in this occurrence.

Several participants stated that they had heard of or had seen others using heroin/fentanyl immediately after being revived with naloxone to mitigate withdrawal symptoms. One participant reported doing this herself, while simultaneously noting that this was “messed up.” Participants reported that people are using in groups as a harm reduction strategy and likened using alone to a death sentence.

### Barriers to Carrying Naloxone Are Primarily Related to Potential Social and Legal Consequences

Participants described several potential barriers when speculating why an individual might choose not to administer naloxone: fear of legal repercussions; not having naloxone available at that moment; and not wanting to interrupt the individual’s euphoric experience (“high”). Interestingly, some participants felt that having naloxone on their person would be perceived by other people as an admission that their recovery might not be successful, and that this decreased their desire to carry it. Of note, two participants expressed concern that carrying naloxone might be interpreted specifically by authority figures (eg, parole officers) as a return to drug use, which would potentially result in legal repercussions. None of the participants had ever self-administered naloxone or knew of anyone who had; all believed that it was impossible or very difficult to do so when indicated.

Good Samaritan laws, which vary by state, protect individuals from prosecution for drug possession if they seek emergency services assistance for a suspected overdose.^11^ Nine participants expressed some understanding of the Good Samaritan Law in Massachusetts, but there was variable comprehension about what this law covers. Several participants also expressed concern over whether law enforcement agencies would adhere to these laws.

### Additional Novel Findings

Most participants shared the belief that the majority/all of the current “heroin” supply in their community is actually fentanyl, and that obtaining “real heroin” was a difficult thing to do. Fentanyl was reportedly less desirable because it was perceived as more dangerous and shorter-acting than heroin, requiring more frequent dosing. Most participants identified cyanosis as the major indicator differentiating the desired opioid effect from an overdose. These participants described the presence of a skin “color change” to blue as the signal to administer naloxone.

## DISCUSSION

Our participants were familiar with and accepting of naloxone. They were also willing to administer this medication to someone who had overdosed. However, participants tended to rely upon the presence of cyanosis, a late finding in overdose, as the indication for naloxone administration. Despite a willingness to carry and use naloxone, we found that some participants associated possession of naloxone with feelings of weakness or potential failure. We found that participants denied engaging in riskier opioid use behaviors when naloxone was available. In fact, some individuals who had previously overdosed and received naloxone held such a strong aversion to the experience of precipitated opioid withdrawal that they reported subsequently using less drug to decrease their overdose risk. Although our data set was small, we did establish thematic saturation for a preliminary study with respect to the question of whether naloxone facilitated riskier drug use: Our participants were unanimous in reporting that they did not decide to use opioids nor increase their opioid use because of increased naloxone availability.

Previous studies have indicated that non-opioid users hold overall positive opinions of naloxone.^12^–^14^ A survey of lay persons found that while only 61% of respondents had heard of naloxone, most respondents (88%) felt naloxone was beneficial in preventing accidental opioid overdoses.^12^ Both medical professionals and state government agencies support efforts to increase naloxone availability due to demonstrated benefits in reducing opioid overdose mortality.^15^,^16^ However, an oft-repeated criticism of naloxone distribution efforts lies in the idea that naloxone availability enables individuals to use opioids without the fear of death, thereby encouraging high-risk drug use behaviors.^17^ A majority of the lay public felt that naloxone was only necessary for people who misuse opioids, and that the availability of naloxone enabled these individuals to increase their opioid use.^12^ Lay media reports have perpetuated the idea of “Narcan parties” or “Lazarus parties,” where people intentionally use large amounts of opioid to overdose with the expectation that they will subsequently be revived by naloxone administration.^4^,^5^

Despite the persistence of these views in popular opinion, our data and the available literature contradict the supposition that enhanced availability of naloxone leads to increased opioid use.^1^,^4^,^18^ Our participants reported no increase in their drug use in spite of widespread availability of naloxone. Instead, they actively attempted to avoid naloxone reversal due to the associated adverse effects and were somewhat reluctant to administer it to others unless they were sure they needed it. Although our sample is small, it consists of a relatively experienced group of people who use opioids, as evidenced by prior treatment attempts for OUD and number of drug-related ED visits. Our preliminary finding that this group did not report adopting riskier drug-use patterns in the context of increased naloxone availability suggests that proliferation of bystander naloxone programs does not beget increased opioid use.

Overall, many of our participants had a high degree of functional knowledge regarding naloxone, held a generally positive view of naloxone, and expressed a willingness to administer naloxone when necessary. Despite traumatic experiences associated with receiving naloxone, participants perceived naloxone as a life-saving medication. Contrary to the popular belief that individuals increase their drug use when naloxone is available, some participants reported that they used less opioids to avoid being administered naloxone. Additionally, our participants described using in groups as a contingency plan to mitigate the risk of overdose, and do not view naloxone as a facilitator of riskier drug use.

Our results suggest several areas that can be targeted to enhance public health interventions. There was widespread thought among participants that the presence of cyanosis (“color change”) in an individual is the primary indicator of overdose and the need for naloxone administration. Future naloxone education efforts targeted to PWUD, as well as the lay public, should stress that cyanosis is a late finding and emphasize indicators that differentiate “high” from overdose, such as shallow or slowed breathing. Our participants suggested that visits to needle exchanges and discharges from treatment programs are high-value times to ensure that PWUD are equipped with naloxone. Furthermore, they identified mobile outreach programs as a desirable community-based harm-reduction service. Public health initiatives should also work to address concerns that carrying naloxone may signal unsuccessful recovery, and instead rebrand bystander naloxone as a willingness to save others’ lives. It may further be beneficial to increase public awareness that naloxone is not for self-administration.

## LIMITATIONS

The limitations of this pilot study include a small sample size (n = 10) and the fact that it was conducted in a single community where several groups were over-represented (eg, male, White, prior naloxone resuscitation, prior treatment for OUD). Although our study population was fairly homogenous and not representative of PWUD on a national scale, it is a typical sample for PWUD in our region in terms of demographics and experience with drug use. We did not appreciate a difference in characteristics between approached vs enrolled patients. Nevertheless, this may detract from generalizability to other settings where the demographics may differ and individuals may have cultural differences or less familiarity with opioid use, opioid antagonists, and treatment modalities for OUD.

That our study was conducted in an urban ED at the epicenter of the North American opioid epidemic likely does skew our study population to favor individuals with more experience and health literacy surrounding their substance use disorder, as evidenced by a majority having previously received naloxone for overdose reversal and treatment for OUD. Furthermore, our state government’s progressive response to the opioid epidemic likely enhances our PWUD population’s familiarity with naloxone. Additionally, although all study staff are trained in qualitative interview techniques, inadvertent use of leading questions could have led to interviewer bias.

Since the data were analyzed by the qualitative interviewers, there was no ability to blind the coders. This could have resulted in bias when assigning the thematic codes, which is why two independent reviewers coded the data and analysis was reviewed by all researchers. Moreover, we were unable to administer the interview in languages other than English, resulting in the exclusion of several individuals who were otherwise eligible for participation. Finally, our study used self-report of an illegal and stigmatized behavior, rather than direct observations of how naloxone availability affected drug use behaviors; the results may therefore be influenced by recall bias and social desirability bias. These factors limit the generalizability of our findings to other demographic groups and locales.

## CONCLUSION

We found that participants were accepting of, knowledgeable about, and willing to use naloxone. Furthermore, we discovered that participants did not increase their use of opioids when naloxone was available, but rather tended to use opioids more cautiously due to fears of experiencing precipitated withdrawal from naloxone administration. These findings further support the need for increasing access of naloxone to help prevent opioid overdose deaths.

## Figures and Tables

**Table 1:** Participant demographics.

	N=10
Median age, years (range)	33 (20–56)
Sex, N	
Male	8
Female	2
Race and ethnicity, N	
White, neither Hispanic nor Latino	8
Native Hawaiian or other Pacific Islander, Hispanic or Latino	1
Multiracial, neither Hispanic nor Latino	1
Married or have significant other, N	
Yes	5
No	5
Number of children, N	
None	5
1–2	3
3+	2
Highest level of education completed, N	
Less than high school	1
High school diploma or equivalent	1
Some college, no degree	5
Associate degree	3
Primary employment status (past 12 months), N	
Unemployed	2
Employed full-time	3
Student	1
Employed full-time and student	1
Retired/disabled	3
Primary housing situation (past 12 months), N	
Homeless	2
Apartment	3
House	3
Sober living house	2
Chief complaint for this ED visit, N	
Suspected opioid overdose, naloxone administered	4
Other opioid-related chief complaint	6
Received naloxone (lifetime), N	
Yes	8
No	2
Been in treatment for substance use (lifetime), N	
Yes	10
No	0
Number of prior drug-related ED encounters (lifetime), N	
None	2
2–5 encounters	5
6+ encounters	3

*ED*, emergency department.

**Table 2:** Illustrative comments from study participants.

Theme	Quotes
Familiarity with Narcan (mechanism and use)	“[Narcan] basically pulls the opiate out of the receptor.”
	“[Narcan is] a reversant of heroin overdose.”
	“I want to help others; I’m not walking around with [Narcan] just for the hell of it; I’m gonna try to save a life.”
Naloxone is available and easy to obtain	“I get [Narcan] for free, I never paid one dollar for it. There’s plenty of programs that give it out for free.”
	“[Narcan]’s not hard to get, so no excuse. Nothing to prevent them from getting it.”
Naloxone availability is viewed positively	“[I] think [Narcan is] an amazing drug. I seen it save people’s lives.”
	“You could probably walk to the corner and you always see someone out [overdosed]...If someone needed [Narcan], I would rather have it than be powerless.”
	“[Narcan is] the best tool to have. It’s the best tool to use.”
Naloxone produces aversive symptoms during reversal	“Nobody wants to be Narcan’d.”
	“[Receiving Narcan feels like] instant withdrawal, but the worst withdrawal you ever felt in your life. Like you feel like your legs are broken, your head’s screaming.”
	“[Receiving Narcan is] kind of embarrassing and degrading and you know it’s upsetting.”
	“[When receiving Narcan for an overdose], it’s better to feel the pain than die.”
The availability of naloxone does not increase risky drug use behavior	“It’s not like we use heroin because we have naloxone...I’ve never seen anyone that wouldn’t already do heroin, do heroin because they have naloxone.”
	“Maybe people are using [heroin] more in groups now because obviously they’re not going to administer naloxone on their dead body.”
Barriers to carrying naloxone are primarily related to potential social and legal consequences	“Trust me, the cops don’t follow the Good Samaritan law. They don’t have to...They’re supposed to, but it doesn’t mean they do.”
• Knowledge of Good Samaritan Laws	“It’s if-if you get caught with somebody, if-if they’re both high and you... are using [drugs], and he is using, [the police] can’t arrest you.”
• Barriers to reviving others	“Say you needed Narcan, and I was gonna be the one to give it, maybe I’d be hesitant ‘cause I’m like, ‘I don’t know, I don’t wanna ruin his high.’”
	“[After giving someone Narcan,] now you have someone who’s sick who wants your dope... So they’re not highly regarded.”
